# Influence of Dental Intervention and Disease on Acute Invasive Fungal Rhinosinusitis Severity and Outcomes

**DOI:** 10.1177/19458924251382514

**Published:** 2025-09-25

**Authors:** Jakob L Fischer, Nikitha Kosaraju, Katherine M Lucarelli, Connie M Sears, Jivianne T Lee, Daniel M Beswick, Daniel B Rootman, Marilene B Wang, Jeffrey D Suh

**Affiliations:** 1 Department of Otolaryngology – Head and Neck Surgery, 8783 University of California, Los Angeles, Los Angeles, California; 2 Department of Surgery, 1685 Uniformed Services University of the Health Sciences, Bethesda, Maryland; 3 Department of Surgery, 21764 University of New Mexico Hospital, Albuquerque, New Mexico; 4 Jules Stein Eye Institute, 547158 University of California, Los Angeles, Los Angeles, California

**Keywords:** acute invasive fungal sinusitis, dental procedures, dental extraction, mucormycoses, rhinoorbital fungal sinusitis, invasive fungal sinusitis

## Abstract

**Background:**

Acute invasive fungal rhinosinusitis (AIFRS) is an aggressive and often fatal disease process that principally impacts immunocompromised patients. Maxillary dental trauma and infections have been associated with the development of maxillary sinus fungal balls, but the role of dental procedures/trauma in the pathogenesis of AIFRS remains poorly defined.

**Objective:**

This study seeks to review a single-institutional experience with AIFRS and examine the association between dental events and AIFRS severity and outcomes.

**Methods:**

Retrospective review of 95 consecutive patients with biopsy-proven AIFRS treated at a tertiary institution between 2010 and 2024. Demographic information, comorbidities, disease course and outcomes were evaluated. The primary objective was to evaluate the impact of antecedent dental events on AIFRS morbidity and mortality. Secondary objectives included evaluating variability in demographic factors, comorbidities, and extent of disease.

**Results:**

Eleven patients with an antecedent dental event within 2 weeks of AIFRS diagnosis were identified for a rate of 11.6%. Dental AIFRS patients were more likely to be African American (*P* = .003) and more likely to have diabetes mellitus as their underlying immunodeficiency (*P* = .03) than non-dental AIFRS patients. Patients with dental-related AIFRS were more likely to present with invasion of the orbit (OR 6.0, 95% CI 1.2-29.5) and nasal floor (OR 4.2, 95% CI 1.1-17.1) than non-dental AIFRS patients. There was no difference in mortality between dental and non-dental AIFRS (36.4% vs 52.4%, *P* = .31).

**Conclusion:**

More investigation is necessary to further evaluate the association between dental events and the development of AIFRS. In our cohort, 11.6% of patients experienced AIFRS within 2 weeks of a dental event and these patients tended to present with higher rates of orbital involvement without a resultant increase in mortality.

## Introduction

Acute invasive fungal rhinosinusitis (AIFRS) is an aggressive, rare disease process that invades paranasal sinuses and nasal cavity with the ability to invade critical adjacent structures such as the orbit and brain.^1^ This life-threatening infection can progress quickly and prove fatal if not aggressively managed.^2^ Mortality rates range from 21% to 80% despite medical and surgical intervention,^3–5^ with a 2013 meta-analysis noting an overall mortality rate of 50.3%.^2^ Patients with AIFRS are almost always immunocompromised at disease onset, with diabetes mellitus (DM2) being the most common etiology, present in 30% to 50% of patients,^6^ but is also seen in patients with other immunocompromising states such as solid organ transplants, liver dysfunction, hematologic malignancies, and AIDS.^2,4,6^

AIFRS is managed in a multimodal fashion with early surgical debridement, systemic antifungal therapy, and reversal of the underlying immunocompromising state. Endoscopic sinus surgery with debridement of necrotic tissues has an established role in AIFRS management with earlier surgical intervention demonstrating increased survival benefit.^2,3^ The goal of surgery is to debride all necrotic tissues that would not otherwise be affected by systemic therapy while eliminating gross disease.^7^ Amphotericin B is the predominant systemic therapy for AIFRS with liposomal formulations demonstrating reduced mortality compared to conventional formulations,^2,8^ although there may be a role of combination or dual antifungal therapy in the management of AIFRS.^9^

Maxillary dental procedures, presumably through oroantral communication, have been associated with the development of maxillary sinus mycetoma/fungal ball,^10^ but the association between dental work and AIFRS is less clear. To date, only case reports^11–14^ and small retrospective reviews^15,16^ have attempted to examine the association between dental events and AIFRS with no studies to date evaluating the impact of these findings on disease severity or patient outcomes. The principal objective of this study was to evaluate the influence of dental disease and procedures on the presentation, extent of disease, and outcomes of patients with AIFRS. Secondary objectives were to evaluate for additional clinical, surgical, and treatment factors that may influence outcomes in this specific subset of patients.

## Methods

A retrospective review of all hospitalized patients at a single tertiary institution with biopsy-confirmed AIFRS between January 2010 and June 2024 was performed. Patients were identified by review of surgical and procedural records and an internal pathology database for all tissue positive cases of invasive fungal rhinosinusitis. Patients identified through the internal pathology database were cross-referenced with medical records and all patients with non-sinonasal invasive fungal infections were excluded. All other patients, independent of their candidacy for operative or procedural intervention, were included in this study cohort. Institutional review board approval was obtained through the University of California, Los Angeles (UCLA) Institutional Review Board (Reference: IRB#24-000341).

**Figure 1.**
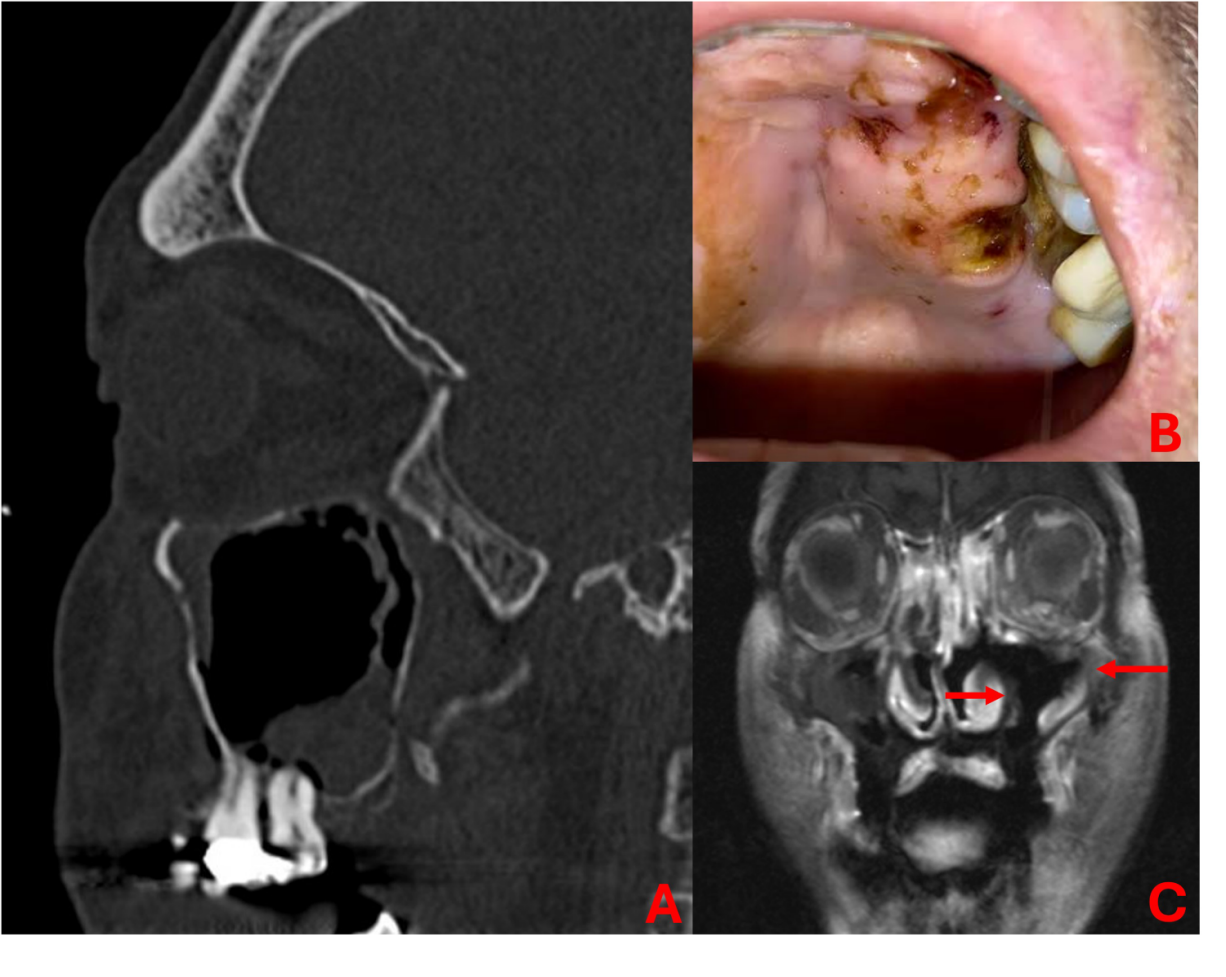
Sagittal CT (A) demonstrating maxillary sinus opacification in setting of dental work. Oral examination (B) at presentation with necrosis of palate and gingival recession adjacent known recent dental work. Coronal post-contrast T1-weighted MRI (C) demonstrating hypoenhancement of mucosa adjacent areas within maxillary sinus (red arrows) concerning for acute invasive fungal rhinosinusitis.

Patient charts were reviewed, and data were extracted. Demographic factors including patient age, sex, race, primary language, ethnicity, comorbidities/risk factors to include hemoglobin A1C (HbA1c), and absolute neutrophil count (ANC) were obtained. Additional information regarding fungal organisms identified on pathologic analysis, systemic therapies utilized, baseline ophthalmologic examination (visual acuity, tonometry, and pupil reactivity), surgical findings, and outcomes of care were included. The surgical extent of disease was determined based on review of operative and pathology reports and any available pre-operative imaging. Operative reports indicated locations of frank necrotic, hypovascular, or fungal disease encountered during endoscopic debridement. Pathology reports were additionally reviewed to determine additional areas of invasive fungal debris by anatomical subsite on final pathologic analysis. All patient charts were thoroughly reviewed to evaluate for antecedent dental events. A dental event was defined as any procedure to include dental extractions, root canal, dental implant placement or removal, fractured teeth, and/or the presence of purulent or erosive dental disease on presentation. Patients with any report of dental events noted in their charts within 30 days of symptom onset with eventual diagnosis of AIFRS were included for final analysis as a dental event. Figure 1 demonstrates an example of the physical exam and imaging findings of a patient with an antecedent dental event.

Patient data was collected in a deidentified database for descriptive statistics and statistical analysis. For descriptive statistics, counts (percentages), means (standard deviation), and medians (quartiles), as appropriate, were reported. Bivariate and multivariate analysis comparisons of mean values were evaluated using either independent, two-tailed t-testing or one-way analysis of variance (ANOVA) for parametric data. *χ*^2^ analysis was performed to evaluate differences in proportions among categorical variables. Type-I error probabilities (*P*-value) are reported for each association of interest. All tests used a significance level of ≤.05.

## Results

Between 2010 and 2024, a total of 95 patients were identified with a tissue-confirmed diagnosis of AIFRS. The average patient was 55.6 ± 16.7 years of age with a slight male predominance (56 males, 59%). White patients represented the largest racial demographic (38, 40%) followed by Asian (8, 8.4%) and Filipino (6, 6.3%) patients. A slight minority of patients identified as Hispanic or Latino (45, 47.4%) and Spanish was the primary language in over a quarter of patients (27, 28%). DM2 was the most common immunocompromising condition (58, 61.1%) followed by leukemia/lymphoma (24, 25.3%) and solid organ transplant (16, 16.8%) (Table 1**)**.

**Table 1.** Patient Demographics.

	Non-Dental Cohort	Dental Event Cohort	Total Population	*P*-Value^a^
Number of patients (*n*, %)	84 (88.4%)	11 (11.6%)	95	
Age (average, SD)	55.0 ± 17.2	59.7 ± 8.8	55.6 ± 16.7	.44
Gender				.11
Male	52 (61.9%)	4 (36.4%)	56 (59%)	
Female	32 (38.1%)	7 (63.6%)	39 (41%)	
Race				**.003**
White	35 (41.7%)	3 (27.3%)	38 (40%)	
African American	1 (1.2%)	3 (27.3%)	4 (4.2%)	
Asian	7 (8.3%)	1 (9.1%)	8 (8.4%)	
Filipino	6 (7.1%)	0 (0%)	6 (6.3%)	
American Indian	1 (1.2%)	0 (0%)	1 (1.1%)	
Indigenous	1 (1.2%)	0 (0%)	1 (1.1%)	
Other	13 (15.5%)	1 (9.1%)	14 (14.7%)	
Unknown	20 (23.8%)	3 (27.3%)	23 (24.2%)	
Ethnicity				.84
Hispanic or Latino	40 (47.6%)	5 (45.5%)	45 (47.4%)	
Not Hispanic or Latino	42 (50.0%)	6 (54.5%)	48 (50.5%)	
Unknown	2 (2.3%)	0 (0%)	2 (2.1%)	
Primary language				.7
English	58 (69.0%)	6 (54.5%)	64 (67.4%)	
Spanish	23 (27.4%)	4 (36.4%)	27 (28.4%)	
Other	3 (3.6%)	1 (9.1%)	4 (4.3%)	
Comorbidities^b^				
Diabetes mellitus	48 (57.1%)	10 (90.9%)	58 (61.1%)	.**03**
Hemoglobin A1C (average, SD)	9.2 ± 3.5	10.2 ± 3.7	10.2 ± 3.4	.56
Diabetic ketoacidosis	23 (27.4%)	4 (36.4%)	27 (28.4%)	.54
Leukemia/lymphoma	23 (27.4%)	1 (9.1%)	24 (25.3%)	.19
Absolute neutrophil count (average, SD)	6.7 ± 6.5	7.3 ± 4.9	6.8 ± 6.3	.56
Solid organ transplant	15 (17.9%)	1 (9.1%)	16 (16.8%)	.47
Liver disease	14 (16.7%)	0 (0%)	14 (14.7%)	.14
End stage renal disease	9 (10.7%)	1 (9.1%)	10 (10.5%)	.87
Length of hospitalization (days) (average, SD)	29.2 ± 32.0	26.8 ± 19.0	29 ± 30.8	.44
Outcome				.31
Alive	36 (42.9%)	5 (45.5%)	41 (43.2%)	
Deceased	44 (52.4%)	4 (36.4%)	48 (50.1%)	
Lost to follow-up	4 (4.8%)	2 (18.2%)	6 (6.3%)	

a Bold values indicate statistically significant results.

b Numbers for individual comorbidities add up to over 100% of the population as some patients had multiple comorbidities. All patients had at least one immunocompromising condition.

Initial chart review identified 13 (13.7%) patients with dental events preceding their diagnosis of AIFRS. Two dental events, one dental implant placement and one dental implant removal, occurred more than 30 days prior to the onset of symptoms and were subsequently excluded from this subgroup, leaving 11 (11.6%) in the final dental event cohort. Of note, all patients within the dental event cohort had antecedent dental work within 14 days of AIFRS symptom onset. Dental events included: 5 (45.5%) dental extractions, 5 (45.5%) cases of dental trauma/infection, and 1 (9.1%) root canal. Within the dental trauma/infection group, 2 patients had poor dentition with multiple fractures, 1 had an abscessed maxillary tooth, 1 had a broken root canal, and 1 had progressive erosion at the gingival border due to poor denture fit. There were no differences in patients with and without dental events in terms of age, gender, ethnicity, or primary language. Patients with an antecedent dental event were more likely to be African American than those without an antecedent dental event (27.3% vs 1.2%, *P* = .003). Patients with antecedent dental events were more likely to be diabetic (90.9% vs 57.1%, *P* = .03) although there was no difference in the percentage of patients presenting in diabetic ketoacidosis (36.4% vs 27.4%, *P* = .54) or in average hemoglobin A1C (10.2 ± 3.7 vs 9.2 ± 3.5, *P* = .56). There were no differences between populations with regards to the presence of other immunocompromising conditions, or length of hospitalization. Mortality was similar between dental event-related AIFRS and non-dental AIFRS (36.4% vs 52.4%, *P* = .31) (Table 1).

Patients presenting with an antecedent dental event had no statistically significant differences in fungal organisms isolated or systemic antifungal treatment. When examining individual anatomical locations of disease, patients with dental-related disease had increased rates of orbital invasion (odds ratio (OR) 6.0, 95% confidence interval (CI) 1.2-29.5) and nasal floor involvement (OR 4.2, 95% CI 1.1-17.1) compared to non-dental event AIFRS patients. There was a non-significantly increased rate of maxillary sinus (OR 4.1, 95% CI 0.83-20.1), pterygopalatine fossa (OR 1.6, 95% CI 0.38-6.7), and inferior turbinate invasion (OR 2.9, 95% CI 0.78-10.4) when comparing dental and non-dental AIFRS (Table 2).

**Table 2.** Fungal and Treatment Characteristics.

	Non-Dental Cohort	Dental Event Cohort	Total Population	Odds Ratio^a^ (95% Confidence Interval)
Number of patients (*n*, %)	84 (88.4%)	11 (11.6%)	95	**–**
Fungal organism isolated				
Rhizopus/Mucor	50 (56.6%)	10 (90.9%)	60 (63.2%)	6.8 (0.83-55.6)
Aspergillus	33 (37.3%)	1 (9.1%)	34 (35.8%)	0.15 (0.02-1.3)
Candida	4 (4.5%)	1 (9.1%)	5 (5.3%)	2.0 (0.20-19.7)
Other species	7 (7.9%)	0 (0%)	7 (7.4%)	0.45 (0.02-8.4)
Multiple Isolates^a^	11 (12.4%)	2 (18.2%)	11 (11.6%)	1.33 (0.26-6.9)
Systemic antifungals in treatment course				
Amphotericin B	75 (89.3%)	11 (100%)	86 (90.5%)	2.9 (0.16 −53.2)
Caspofungin	39 (46.4%)	5 (45.5%)	44 (46.3%)	0.96 (0.27-3.4)
Posaconazole	36 (42.9%)	3 (27.3%)	39 (41.1%)	0.50 (0.12-2.0)
Voriconazole	20 (23.8%)	2 (18.2%)	22 (23.2%)	0.64 (0.13-3.2)
Isavuconazole	45 (53.6%)	7 (63.6%)	52 (54.7%)	1.5 (0.41-5.6)
Micafungin	12 (14.3%)	2 (18.2%)	14 (14.7%)	1.2 (0.23-6.17)
Antifungals per patient (average, SD)	2.7 ± 0.75	2.7 ± 1.0	2.7 ± 1.0	
Total number of surgeries (median, IQR)	2 IQR = 2	2 IQR = 3	2 IQR = 2	
Sites of disease involvement				
Maxillary sinus	44 (52.4%)	9 (81.8%)	53 (55.8%)	4.1 (0.83-20.1)
Ethmoid sinus	40 (47.6%)	4 (36.4%)	44 (46.3%)	0.63 (0.17-2.3)
Sphenoid sinus	27 (32.1%)	3 (28.3%)	30 (31.6%)	0.79 (0.19-3.2)
Frontal sinus	12 (14.3%)	1 (9.1%)	13 (13.7%)	0.60 (0.7-5.1)
Middle turbinate	35 (41.7%)	7 (63.6%)	42 (44.2%)	2.5 (0.67-9.0)
Superior turbinate	8 (9.5%)	0 (0%)	8 (8.4%)	0.39 (0.02-7.2)
Inferior turbinate	19 (22.6%)	5 (45.5%)	24 (25.3%)	2.9 (0.78-10.4)
Pterygopalatine fossa	16 (19.0%)	3 (27.3%)	19 (20%)	1.6 (0.38-6.7)
Infratemporal fossa	6 (7.1%)	2 (18.2%)	8 (8.4%)	2.9 (0.51-16.5
Nasal septum	27 (32.1%)	2 (18.2%)	29 (30.5%)	0.42 (0.09-2.1)
Nasal floor	10 (11.9%)	4 (36.4%)	14 (14.7%)	**4.2** (**1.1-17.1)**
Orbit	36 (42.9%)	9 (81.8%)	45 (47.4%)	**6.0** (**1.2-29.5)**
Skull base	14 (16.7%)	3 (27.3%)	17 (17.9%)	1.9 (0.44-8.0)

a Bold values indicate statistically significant results.

In total, 45 patients (47.4%) had orbital involvement relating to their AIFRS, including 9 (81.8%) patients with an antecedent dental event and 36 (42.9%) patients with no antecedent dental event. At presentation, 55.5% (*n* = 5) of patients with an antecedent dental event had no light perception on baseline examination, compared to 36.1% (*n* = 13) in patients without an antecedent dental event (*P* = .29). The minority of patients in the dental and non-dental groups had any meaningful visual recovery post-treatment, with only 1 (11.1%) patient in the dental event group and 4 (11.1%) in the non-dental event groups having any reported improvements in vision or diplopia. There were no differences in rates of visual recovery between those patients with or without an antecedent dental event (Table 3).

**Table 3.** Orbital Presentation and Outcomes.

	No Dental Event (*n* = 84)	Dental Event (*n* = 11)	*P*-Value^a^
Orbital involvement	36 (42.9%)	9 (81.8%)	**.015**
Visual acuity in affected eye at presentation			.95
Vision 20/100 or better	12 (33.3%)	2 (22.2%)	.52
Vision 20/100 to 20/200	2 (5.6%)	0 (0%)	.72
Vision 20/200 to 20/800	3 (8.3%)	0 (0%)	.37
Limited to count fingers	2 (5.6%)	0 (0%)	.72
Limited to pinhole perception	2 (5.6%)	0 (0%)	.72
No light perception	13 (36.1%)	5 (55.5%)	.29
No baseline acuity reported	2 (5.6%)	2 (22.2%)	–
Patients with elevated intraocular pressure (IOP > 21) (*n*, %)	8 (22.2%)	0 (0%)	
			.12
Pupil reactivity in affected eye at presentation			
			.2
Normal reactivity	15 (41.7%)	1 (11.1%)	.08
Sluggish	6 (16.7%)	3 (33.3%)	.27
Nonreactive	12 (33.3%)	4 (44.4%)	.54
Not obtained	3 (8.3%)	1 (11.1%)	.79
Orbital outcome			
Improved			1
Vision	3 (8.3%)	1 (11.1%)	
Diplopia	1 (2.7%)	0 (0%)	
Unchanged			.33^b^
No vision	4 (11.1%)	4 (44.4%)	
Baseline decreased vision	1 (2.7%)	0 (0%)	
Further visual deterioration	1 (2.7%)	0 (0%)	
Alive with orbital exenteration	5 (13.9%)	0 (0%)	
Patient deceased	21(58.3%)	4 (44.4%)	.31

a Bold values indicate statistically significant results.

b Includes patients with unchanged vision post-treatment, those with further visual deterioration, and those patients undergoing exenteration.

## Discussion

AIFRS is a rare but aggressive disease process with a highly variable presentation. In this study, we sought to examine a subset of patients that presented with AIFRS in the setting of a preceding dental event. Patients with dental-related AIFRS had 6.0 (9.5% CI 1.2-29.5) times increased odds of orbital invasion and 4.2 (95% CI 1.1-17.1) times increased odds of nasal floor involvement compared to non-dental patients. Additionally, patients with a preceding dental event were more likely to be African American and have DM2 as their underlying immunodeficiency. There was no difference in mortality between patient subgroups.

To the best of our knowledge, this is the largest study to specifically examine the potential role of dental events in a large cohort of patients with AIFRS. The current literature investigating the role of dental events in the pathogenesis of AIFRS is limited to a small series of retrospective reviews and case reports. Most recently, Li et al examined seven patients with oral and maxillofacial mucormycoses over an 8-year period, identifying three diabetic patients who reported maxillary dental extractions in the days preceding their AIFRS diagnosis. A concurrent literature review examined an additional 48 articles with a total of 88 patients with craniomaxillofacial AIFRS and found that up to 31.8% of patients had disease present after dental extraction.^15^ Interestingly, this rate is over double that identified in the current study's patient population (11.6%). This difference in dental-related AIFRS rates is likely at least in part due to the current study being limited to fungal rhinosinusitis while their literature review included patients with mandibular disease as well. A second retrospective review, performed by Emodi et al, identified 10 patients with mucormycoses treated over an 11-year period. Of these patients, 4 (40%) were noted to have undergone maxillary dental extractions prior to diagnosis, although the interval between extraction and diagnosis is not reported.^16^ In contrast to the patients in this study and the other retrospective review, all patients in the Emodi et al cohort were diagnosed with leukemia/lymphoma with a resultant higher reported mortality (75%) within their dental-related patient cohort.^16^ In the only U.S. case series examining patients with dental-related AIFRS, Kim et al examined 4 patients over a 10-year period that presented with AIFRS within days of dental extractions. All patients presented with uncontrolled diabetes and rapidly progressive orbital findings with eventual mortality in 3 patients.^14^

While this study and those studies cited above note that between 13% and 32% of AIFRS cases may be related to preceding dental procedures or pathology, causality cannot be established in a retrospective review alone and further study would be required to imply causality. Despite this limitation, there is a known risk of bacteremia associated with tooth brushing (up to 23%) and single tooth extraction with (33%) and without (60%) antibiotics as based on a double-blind placebo-controlled trial of 290 patients with serial blood cultures,^17^ but the role of dental associated fungemia and local fungal burden is less clear. Debelian et al examined the microbiome within the apical tooth roots of patients undergoing root canals and compared these findings to blood cultures in 26 adult patients and isolated 132 local microbial strains with a 54% yield of cultivable microorganisms within the blood stream, including fungi such as *saccharomyces cerevisiae,* indicating the potential for fungemia related to dental procedures.^18^ Damasceno et al, noted the presence of biofilms in water samples obtained from two French dental clinics that containing filamentous fungi, including those with the potential to cause AIFRS. They concluded that these water sources had the potential to aerosolize fungi which may represent a potential risk, especially to immunocompromised patients undergoing dental treatment.^19^

While additional investigation is necessary to further elucidate the association between dental procedures and the development of AIFRS; this may highlight the need for continued optimization of medical care in these immunocompromised patient populations. Interestingly, most studies demonstrated a significant preponderance of dental-related AIFRS in patients with DM2, as opposed to other immunocompromising conditions. This may be in part due to the screening and recommendations associated with the management of other immunocompromising conditions such as malignancies. The National Cancer Institute recommends that patients undergo dental evaluation 4 weeks prior to initiating cancer treatment to allow for adequate healing should dental work be required or performed between cycles of therapy to minimize the patient's immunocompromised state during active treatment.^20^ These recommendations may in part explain the lower prevalence of dental-related AIFRS in our study population relative to DM2, where the patient's immunocompromised status may not be as readily apparent.

As this study is a retrospective analysis of a rare disease process with patients spanning 14 years, it is subject to several limitations. First, the sample size is comparatively small, particularly for the cohort that experienced dental-related AIFRS. This limits the ability to perform multivariate analyses and results in wide confidence intervals that may increase the risk of type 2 error and results in relatively large confidence intervals. As this is a retrospective review examining the association between dental events and AIFRS, there is also a possibility that patients may not have been thoroughly screened for antecedent dental events and there may have been dental events not in our study cohort, particularly as dental records are not routinely available for review in our electronic medical record. Our study examines nearly 15 years of data at a single institution, and this inherently introduces some bias to our patient populations and adds inherent variability to the work-up, diagnosis, and treatment of individual patients. Advances in medical therapy, particularly the management of immunocompromising conditions, the management of AIFRS with systemic medications, and our improved recognition of AIFRS potentially influences our patient outcomes to a degree that cannot be accounted for in our study design and may impact overall mortality. These limitations may be aided by future review with larger population sizes and multi-institutional studies.

## Conclusion

AIFRS is a rare, rapidly progressive, and often fatal disease process. Patients with an antecedent dental event prior to diagnosis of AIFRS had higher odds of nasal floor invasion and orbital invasion that did not portent an increased risk in overall mortality. It may be beneficial to screen patients presenting with AIFRS for antecedent dental event as these patients may present with more advanced disease. More research is necessary to further evaluate these associations.
